# Diagnosis of periprosthetic loosening of total hip and knee arthroplasty using ^68^Gallium-Zoledronate PET/CT

**DOI:** 10.1007/s00402-024-05562-5

**Published:** 2024-10-01

**Authors:** A. Touet, S. Koob, S. Kürpig, J. Roos, F. Roesch, DC. Wirtz, M. Essler, FC. Gaertner

**Affiliations:** 1 Clinic for Orthopedics and Trauma Surgery, University Hospital of Bonn, Venusberg-Campus 1, 53127 Bonn, Germany; 2 Clinic for Nuclear Medicine, University Hospital of Bonn, Bonn, Germany; 3 Institute of Nuclear Chemistry, Johannes Gutenberg-University Mainz, Mainz, Germany

**Keywords:** Periprosthetic loosening, Gallium-Zoledronate, PET/CT, Arthroplasty

## Abstract

**Purpose:**

Periprosthetic loosening is a major complication after total hip and knee arthroplasty. Early and accurate diagnosis is essential to choose the right therapeutic path and to avoid further complications. The aim of the study was to evaluate the diagnostic performance of ^68^Gallium-Zoledronate ([^68^Ga]Ga-DOTA^Zol^) PET/CT in detecting periprosthetic loosening in total hip (THA) and total knee arthroplasty (TKA).

**Methods:**

This retrospective study included 26 patients with painful prosthesis (THA n = 17; TKA n = 16) and clinical suspicion of periprosthetic loosening, but without a confirmed diagnosis. Patients underwent [^68^Ga]Ga-DOTA^Zol^ PET/CT at least one year post-implantation. Diagnosis was confirmed through revision surgery or long-term clinical follow-up, with an observation period of at least 6 months. The analysis included both an assessment of the prosthesis as a unit and a separate evaluation of the individual components. Statistical analysis involved calculating sensitivity, specificity and accuracy using SPSS.

**Results:**

Overall, a sensitivity of 77.8%, a specificity of 95.8% and an accuracy of 90.9% were found for detecting periprosthetic loosening, when considering the prosthesis as a unit. Individual component analyses showed a sensitivity of 71.4% and specificity of 96.2%.

**Conclusion:**

The use of [^68^Ga]Ga-DOTA^Zol^ PET/CT in periprosthetic loosening is a remarkable diagnostic tool and a promising approach. In comparison to established radionuclide tracers, ^68^Gallium-Zoledronate offers notable advantages due to its availability via ^68^Ge/^68^Ga-generators, improving its potential for clinical application.

**Supplementary Information:**

The online version contains supplementary material available at 10.1007/s00402-024-05562-5.

## Introduction

Periprosthetic loosening, both septic and aseptic, is a major long-term complications in total knee (TKA) and hip (THA) arthroplasty, requiring effective diagnostic and treatment strategies [1, 2]. According to the German Arthroplasty Registry, periprosthetic loosening is the most common reason for revision surgery with 22.7% in THA and 22.8% in TKA, ahead of other complications such as infections, periprosthetic fractures and dislocations [2]. Current literature suggests that periprosthetic loosening is primarily caused by a loss of biological fixation due to particle-induced osteolysis around the implant. Pathophysiologically, this is characterized by chronic inflammatory processes with an increased release of macrophages at the bone/prosthesis or cement/prosthesis interface. [3–5]

Early and accurate diagnosis of periprosthetic loosening is crucial for optimal therapy selection and preserving bone stock. However, the sensitivity and specificity of established radiological methods such as native radiographs and bone scans differ greatly [6]. This is primarily explained by micromovements in the early phase that cannot be detected using conventional radiological diagnostics [3, 4]. It is therefore necessary to evaluate alternative or improvement to already established procedures. PET/CT has shown itself to be a promising nuclear medical diagnostic tool, offering combined functional-metabolic and structural imaging with high spatial resolution. [^18^F]fluoride, a commonly used positron-emitting bone-seeking PET tracer, has already shown impressive results in several studies in detecting periprosthetic loosening [6, 7]. Consideration of alternative radionuclides is increasingly important, given tracer availability and clinical requirements. Radiopharmaceuticals labelled with ^68^Gallium, a positron emitter, have become increasingly important in clinical practice and research, due to their favourable physical properties (t½ = 67.8 min; β^+^ yield = 89.1%; E^β−max^ = 1.9 MeV)[8, 9]. The pharmacokinetics and efficacy of ^68^Gallium labelled and ^177^Lu-labelled DOTA^Zol^ compounds have already been confirmed in numerous in vitro and in vivo studies [10–13].

The aim of this study was to evaluate the diagnostic performance of [^68^Ga]Ga-DOTA^Zol^ PET/CT in detecting periprosthetic loosening in patients with total hip (THA) and knee arthroplasty (TKA) at least 1 year after prosthesis implantation. Therefore, the study compared the results of [^68^Ga]Ga-DOTA^Zol^ PET/CT with intraoperative findings and long-term clinical follow-up with an observation period of at least 6 months. To our knowledge, this is the first analysis on the use of [^68^Ga]Ga-DOTA^Zol^ PET/CT in this context of periprosthetic loosening.

## Material and methods

This retrospective study included 26 patients with painful total hip (THA; n = 17) and total knee arthroplasty (TKA; n = 16) with clinical suspicion of peri-prosthetic loosening. The collective included 12 female and 14 male patients with a mean age of 64.4 years (SD 10.6; range 41–83). Regarding the primary endpoint of this study, the following requirements were placed on the cases to be evaluated: None of the patients had been definitively diagnosed before the [^68^Ga]Ga-DOTA^Zol^ PET/CT was performed. In all cases, [^68^Ga]Ga-DOTA^Zol^ PET/CT was carried out at the earliest 1 year after prosthesis implantation. Patients with an underlying oncological disease were excluded. All patients were subject to routine clinical examination, laboratory, and radiological studies before the [^68^Ga]Ga-DOTA^Zol^ PET/CT scan was performed. All patients included were examined in between 11/2017 and 02/2021.

The final evaluation and diagnosis of periprosthetic loosening was performed through revision surgery or a clinical follow-up. Revision surgery, as preferred path for diagnostic confirmation, was performed by an experienced surgeon 27.1 d (SD 33.7) after PET/CT. Information regarding the fit of the individual prostheses’ components was extracted from the surgical report. In case no revision surgery was performed a long term clinical follow up, with at least 6 months observation time, was used to confirm or disprove the diagnosis. Due to the high image resolution of the PET/CT, separate analysis of the acetabular, femoral and tibial components was possible and findings were assessed as true positive (TP), true negative (TN), false positive (FP) and false negative (FN) according to the intraoperative findings or clinical follow up. We performed two separate analyses: First, the prosthesis was considered as a coherent unit without distinguishing between the hip, femoral or tibial components. Second, each prosthetic component was evaluated individually.

Considering the PET/CT scan protocols, additional asymptomatic knee and hip prostheses shown in the image section were included for further assessment. Five patients had a total of 2 inserted prostheses and one patient had 3 prostheses. The study was approved by the local Ethics Committee (Reg. No. 97/17) and a written declaration of consent from patients for the anonymous use of their clinical data was available.

**Fig.1:**
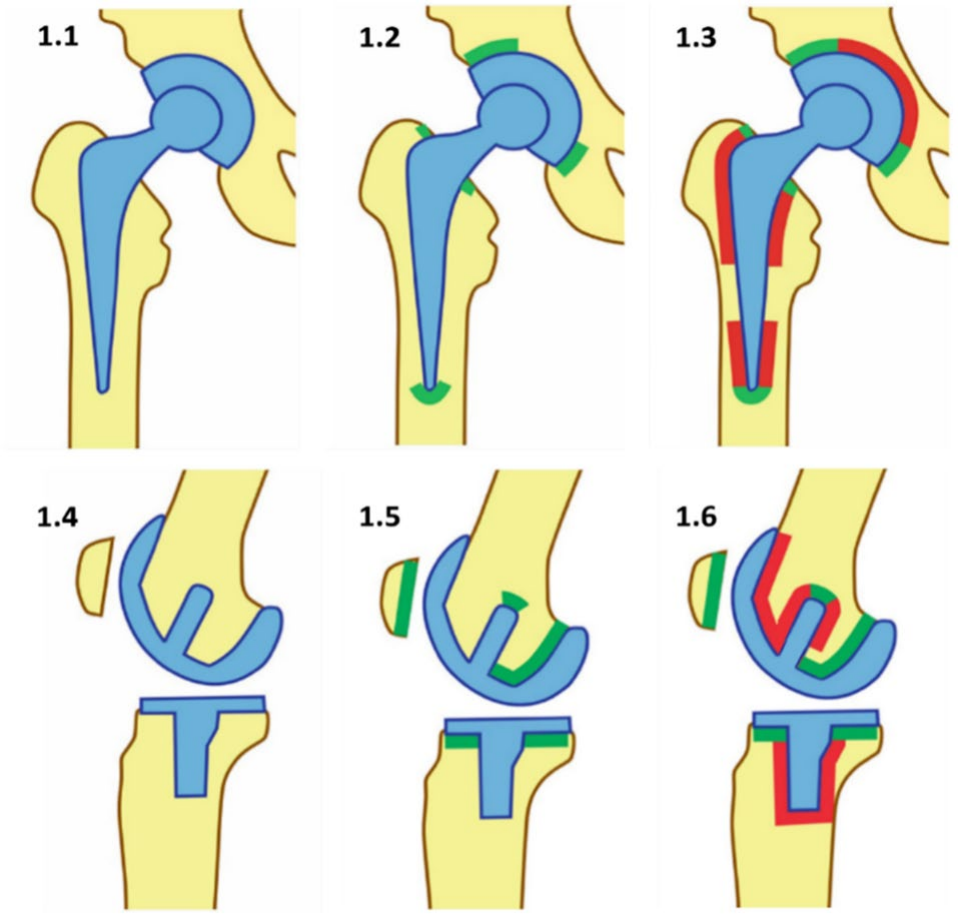
A schematic representation of [^68^Ga]Ga-DOTA^Zol^ uptake pattern (modified from Koob et al.). The unspecific uptake patterns are shown in green, while the areas marked in red were regarded as specific to implant loosening

**Fig. 2:**
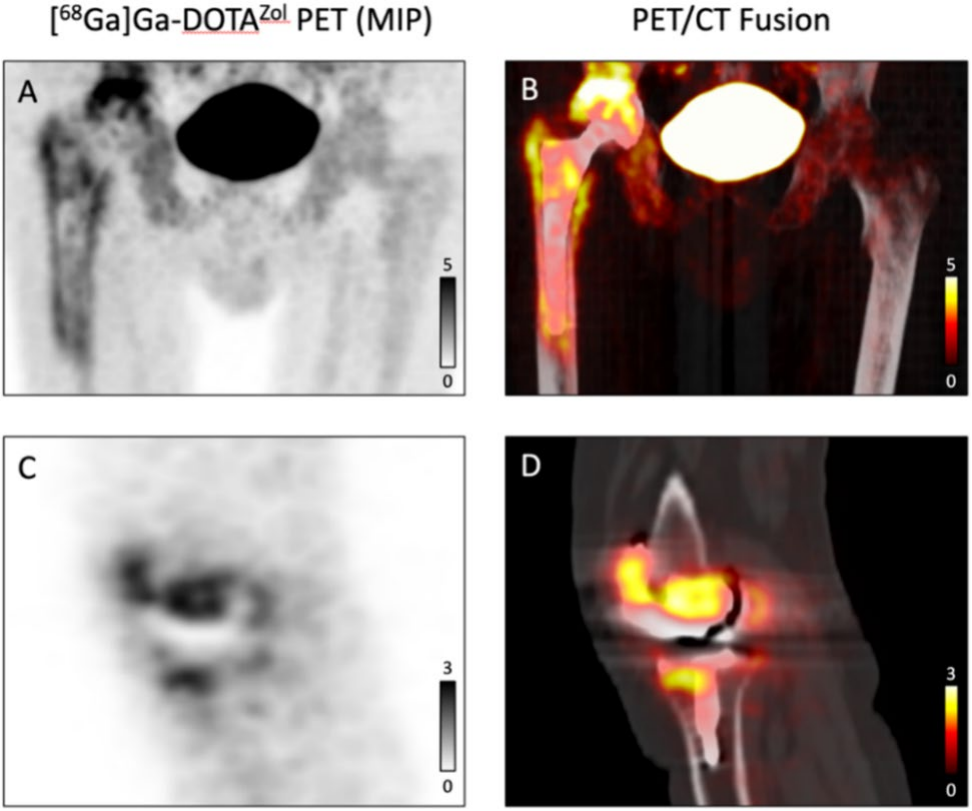
Examples of true positive cases. **A** and **B** True positive case of THA loosening. Tracer uptake is observed at the proximal and distal tips of the femoral component, extending along the whole bone prosthesis interface of the femoral component. **C** and **D** True positive case of TKA loosening. Tracer uptake is observed along the whole bone prosthesis interface of the femoral sled, extending from the dorsal region up to the anterior parts

**Fig. 3:**
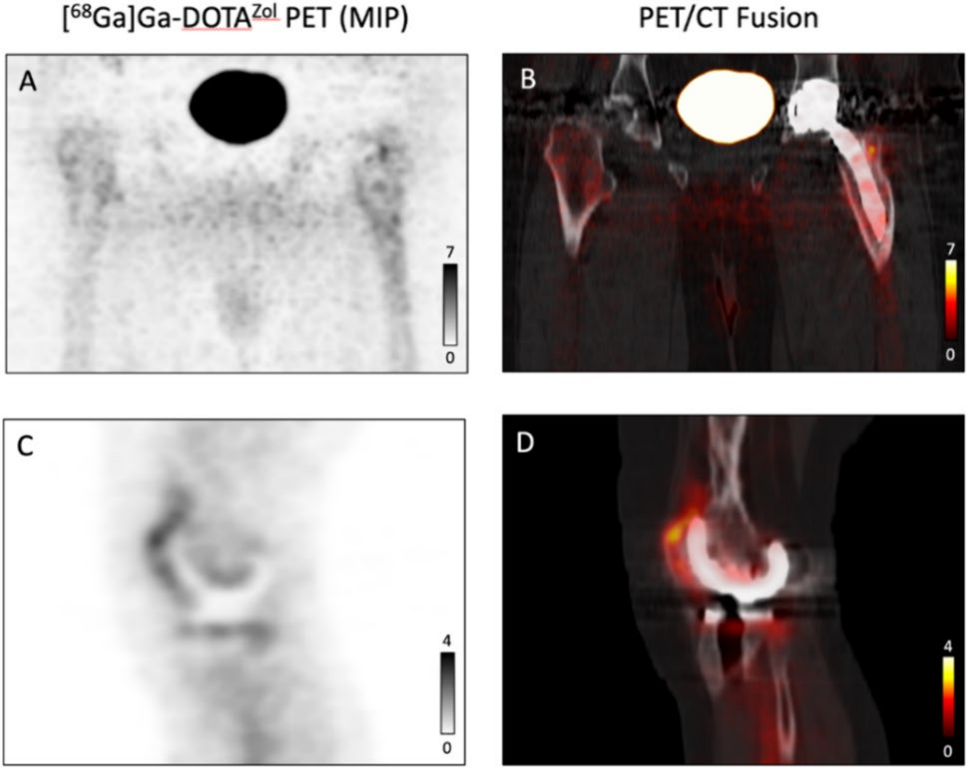
Examples of true negative cases. **A** and **B** True negative case of THA loosening. No significantly elevated

**Fig. 4:**
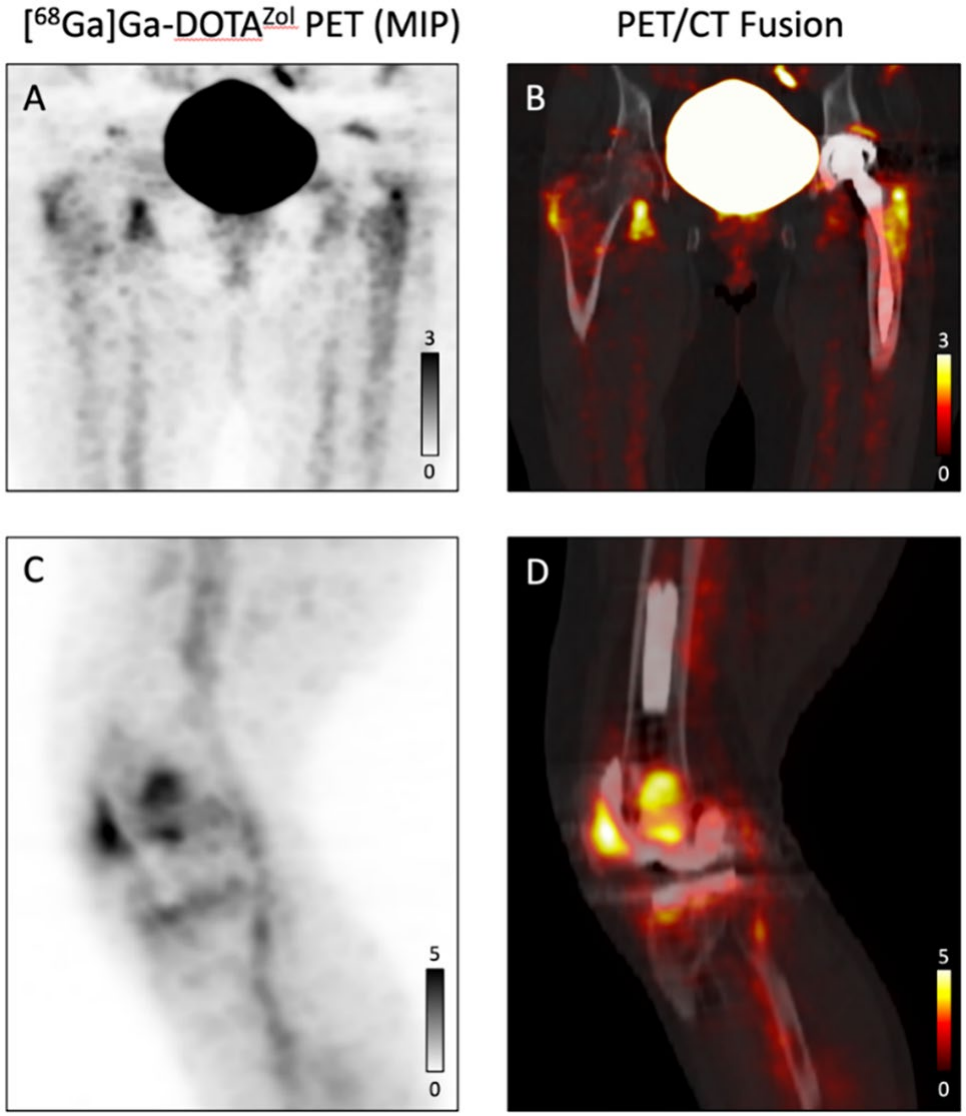
**A** and **B** False positive case of THA loosening. Tracer uptake is observed at the proximal tip of the femoral component, extending down along the bone prosthesis interface of the femoral component, which was falsely rated as positive for loosening. **C** and **D** False negative case of TKA loosening. Tracer uptake is observed at the patellar region, the caudal region of the femoral sled, the tip of the femoral pin and the horizontal tibial surface, which was falsely rated as negative for loosening

Gallium-68 was obtained from a 1.85 GBq ^68^Ge/^68^Ga-generator (GalliaPharm EZAG, Berlin Germany). PET imaging was performed on a Biograph 2 PET/CT scanner (Siemens Healthcare GmbH, Erlangen, Germany). A native low-dose spiral CT was made for attenuation correction and morphological assignment of the emission data and linked to the PET files. Quantitative evaluation of the attenuation corrected data was performed using SUV calculation and reconstruction in axial, coronal, and sagittal planes. The PET/CT scans were evaluated by an experienced nuclear medicine physician, based on the specific enrichment pattern of the [^68^Ga]Ga-DOTA^Zol^ tracer at the prosthesis/bone or cement/bone interface. Following the methodological approach of Koob et al. [6], enrichments in the cranial and caudomedial acetabular component as well as the proximal and the distal outermost tip of the femoral component were evaluated as nonspecific in THA. In patients with TKA, accumulation in the dorsal and caudal areas of the femoral sled, the tip of the femoral pin, the patellar region and the horizontal surface of the proximal tibial component were defined as nonspecific. In contrast, increased enrichment beyond the defined regions was always considered specific and thus indicative of periprosthetic loosening. A schematic representation of the [^68^Ga]Ga-DOTA^Zol^ uptake patterns in THA and TKA is shown in Fig. 1. Selected patient-related case examples are shown in Figs. 2, 3, 4***.***

As outlined above, each case was categorized as true positive (TP), true negative (TN), false positive (FP) or false negative (FN) based on the findings. Regarding the primary endpoint, sensitivity, specificity and accuracy were calculated to evaluate the diagnostic performance of [^68^Ga]Ga-DOTA^Zol^ PET/CT in detecting periprosthetic loosening. The collected data were entered in Excel and transferred to SPSS for further calculation.

## Results

The sample studied included 26 patients with a total of 33 prostheses and accordingly 66 components (tab. 1). The mean time between implantation of the knee or hip prosthesis and PET/CT diagnostics was 6.3 years (SD 6.6). In 9 patients (34.6%) and 9 prostheses (27.3%; 18 components), the [^68^Ga]Ga-DOTA^Zol^ PET/CT diagnosis was confirmed or rejected by revision surgery and intraoperative findings. For 24 prostheses the PET/CT diagnosis was evaluated in a long-term clinical follow-up. One patient with clinically and radiologically/nuclear medicine highly suspected loosening, refused the surgery we recommended (Table. 1; patient 10). Furthermore, one patient did not undergo surgery due to his severe previous cardiac disease (Table. 1; patient 4). Both cases were confirmed as true positive (TP) based on subsequent clinical and radiological assessments.

**Table 1:** Included patients with total hip arthroplasty (THA) and total knee arthroplasty (TKA)

Patient	Prosthesis	Age (y)	Sex	Joint	Result of[68 Ga]GaZol PET/CT	Follow-up	Acetabular Component	Femoral Component	Tibial Component
1	1	56	F	**THA (right)**	No loosening	Clinical	–	TN	TN
	2			THA (left)	No loosening	Clinical	TN	TN	–
2	3	78	F	**THA (left)**	No loosening	Surgery	FN	TN	–
	4			THA (right)	No loosening	Clinical	TN	TN	–
3	5	63	M	**THA (right)**	No loosening	Clinical	TN	TN	–
4	6	70	M	**THA (left)**	Acetabular and femoral loosening	Clinical	TP	TP	–
5	7	48	F	**THA (left)**	No loosening	Surgery	TN	TN	–
	8			TKA (right)	No loosening	Clinical	TN	TN	–
6	9	58	M	**TKA (left)**	No loosening	Clinical	–	TN	TN
7	10	65	F	**TKA (left)**	Femoral loosening	Surgery	–	TP	TN
8	11	57	M	**TKA (left)**	No loosening	Clinical	–	TN	TN
	12			TKA (right)	No loosening	Clinical	–	TN	TN
9	13	67	M	**THA (left)**	Femoral and tibial loosening	Surgery	–	TP	TP
10	14	83	M	**THA (left)**	Acetabular andfemoral loosening	Clinical	TP	TP	–
11	15	75	F	**THA (left)**	Acetabular andfemoral loosening	Surgery	FP	FP	–
	16			TKA (right)	No loosening	Clinical	TN	TN	–
	17			THA (left)	No loosening	Clinical	–	TN	TN
12	18	69	M	**THA (right)**	No loosening	Surgery	–	FN	FN
13	19	74	F	**THA (right)**	No loosening	Clinical	TN	TN	–
14	20	74	M	**THA(right) **	Femoral loosening	Surgery	FN	TP	–
15	21	61	F	**THA (left)**	Femoral loosening	Surgery	TN	FP	–
16	22	78	F	**THA (right)**	No loosening	Clinical	TN	TN	–
17	23	60	F	**TKA (left)**	No loosening	Clinical	TN	TN	–
18	24	48	F	**TKA (left)**	No loosening	Clinical	–	TN	TN
19	25	60	M	**TKA (left)**	No loosening	Clinical	–	TN	TN
20	26	73	M	**TKA (left)**	No loosening	Clinical	–	TN	TN
21	27	60	M	**TKA (left)**	No loosening	Clinical	TN	TN	–
22	28	56	M	**TKA (left)**	No loosening	Clinical	TN	TN	–
23	29	79	F	**TKA (left)**	No loosening	Clinical	TN	TN	–
24	30	58	F	**TKA (right)**	No loosening	Clinical	TN	TN	–
	31			THA (left)	No loosening	Clinical	TN	TN	–
25	32	64	M	**TKA (left)**	No loosening	Clinical	–	TN	TN
26	33	41	M	**THA (left)**	No loosening	Surgery	–	TN	TP

*FN* = false negative, *FP* = false positive, *TN* = true negative, *TP* = true positive, *bold* = clinically suspicious prosthesis

Results revealed a sensitivity of 77.8%, a specificity of 95.8% and an accuracy of 90.9% for prostheses as a coherent unit, yielding a positive predictive value of 87.50%. The dataset included clinically silent prostheses (n = 7; comp. = 14), classified as true negative (TN) based on the negative enhancement pattern in PET/CT and inconspicuous clinical follow-up. Excluding these cases and focusing only on painful prostheses, the sensitivity was 77.8%, specificity lowers to 94.1% and accuracy to 88.5%. Analyzing the individual prosthesis components separately, the sensitivity was 71.4%, the specificity 96.2% and the accuracy 90.9%.

[^68^Ga]Ga-DOTA^Zol^ PET/CT correctly identified 7 out of 9 loosened hip and knee prostheses (RP). For 1 out of 24 prostheses without signs of loosening in the revision surgery or long-term clinical follow-up the PET/CT showed a positive enhancement pattern (FP). 2 out of 25 prostheses with no signs of periprosthetic loosening in the PET/CT ultimately turned out to be loosened due to intraoperative findings or clinical follow-up, classifying them as false negative (FN).

## Discussion

Painful knee and hip prostheses due to periprosthetic loosening, especially aseptic loosening, are common and represent a great challenge for the treating clinician. Accurate diagnostics is essential as the clinical consequence means explantation of the prosthesis and reimplantation, which is associated with increased complications [14, 15]. However, there’s still no consensus on the optimal diagnostic approach, complicating the assessment and classification of new procedures.

In the study, [^68^Ga]Ga-DOTA^Zol^ PET/CT showed a promising diagnostic performance for detecting periprosthetic loosening, with a sensitivity of 77.8% and a specificity of 95.8%. Despite consideration individual prosthesis components, the discussion primarily focuses on the prosthesis as a coherent unit. This corresponds to daily clinical practice, in which revision surgery is considered if loosening of a component is suspected. The direct comparison to other nuclear medical diagnostic tools reveals a heterogeneous picture with partly large variations. In a meta-analysis on aseptic THA loosening, the classic and established method of bone scintigraphy showed an overall sensitivity of 85% (95% CI 79 to 89) and a specificity of 72% (95% CI 64 to 79) [16]. Whereas the meta-analysis on loosened TKA reported sensitivities between 76 and 100% and specificities between 33 and 100% using scintigraphy [17]. Focusing on PET diagnostics, Mayer-Wagner et al. [18] found a comparable sensitivity of 80% for the use of [^18^F]FDG-PET in THA, with a decreased specificity of 87%. TKAs showed a markedly reduced sensitivity of 56% and a specificity of 82%. In contrast, Sterner et al. [7] reported a sensitivity of 100% and an overall poor specificity of only 55% using PET diagnostic with the bone seeking tracer [^18^F]fluoride. For the combined diagnostic procedure of [^18^F]fluoride PET/CT, Koob et al. [6] demonstrated a higher sensitivity of 95% with a reduced specificity of 87%.

Considering the deviating results, the question arises to what extent these can be attributed to methodological approaches, individual sample characteristics or to process/nuclide-specific properties. Thus, Gallium-68 is characterized by a higher positron energy (*E *^*β−*max^:1.9 MeV), compared to Flouride-18. The higher kinetic energy is associated with a poorer spatial resolution and a higher susceptibility to artifacts [19–21]. Accordingly, a reduced detection sensitivity is described in the literature for ^68^Ga-PET compared to ^18^F-PET [22]. However, differences in tracer binding and accumulation properties can mitigate this effect.

The combination of a macrocyclic chelator with a bisphosphonate in [^68^Ga]Ga-DOTA^ZOL^ provides high complex stability and its radiosynthesis is a straightforward process using conventional labelling kits. Zoledronate shows excellent and superior accumulation in bone compared to other bisphosphonate, resulting in a significantly improved blood to bone ratio [10, 23]. After intravenous injection [^68^Ga]Ga-DOTA^ZOL^ shows a comparable activity to [^18^F]NaF, with a slightly slower renal clearance. Thus, [^68^Ga]Ga-DOTA^ZOL^ presents itself as an favourable tracer with highly selective uptake in bone lesions [11].

When discussing the methodological approach, the distinction between non-specific and specific enrichment, along with the timing of the PET/CT is crucial. No respective studies have jet been published for [^68^Ga]Ga-DOTA^Zol^. Based on a comparable biodistribution [10, 11] and presumed similar pathophysiological mechanism, we relied on the criteria already published for [^18^F]NaF [6, 24]. Following the assumption that increased uptake can be taken as an indicator of periprosthetic loosening, the analysis followed the criteria outlined in the methodology section. The determining factor in this case is the enrichment pattern and not solely the intensity of radionuclide uptake [7]. It is well known that increased metabolic activity, with a correspondingly greater accumulation at the prosthesis/bone or cement/bone interface, can occur postoperatively. Following the hypothesis that nonspecific enhancement normalizes after 9–12 months, [^68^Ga]Ga-DOTA^Zol^ PET/CT was performed in our study at least 1 year after implantation to minimize false-positive (FP) results [25]. We observed only one false positive result (prosthesis 3%), which is significantly lower than the findings of comparable studies. However, there are considerable differences in biodistribution properties and tissue uptake mechanisms between the various radiotracers. For example [^18^F]FDG, as a frequently used PET tracer, can show an increased and persistent non-specific uptake over several years after prosthesis implantation [26]. Overall, this demonstrates the need for an exact tracer-specific definition of specific and non-specific enrichment.

Considering revision surgery as the preferred path to confirm the diagnosis, the low percentage of 34% symptomatic prostheses in the surgical control group must be critically questioned. Furthermore, it is important to mention the heterogeneous patient population. This includes PET/CT scans at different time points (> 1 year) after implantation, divergent time intervals between PET/CT diagnostics and control examinations as well as scans of various prostheses. In this context, Ullmark et al., using [^18^F]fluoride PET, found a significantly higher and also prolonged metabolism for patients with uncemented THA compared to cemented stems [27]. However, very few data are available on this, and a dedicated analysis regarding this effect was not possible in the present study due to the limited sample size. Nevertheless, this reflects everyday clinical practice, even if the present patients are inconclusive cases. Patients with clinically or radiologically clear signs of periprosthetic loosening did not undergo further diagnostics (PET/CT). Accordingly, the results must be evaluated differently since pre-test probability rises.

Another crucial aspect is the differentiation between aseptic and septic loosening. Even with a different underlying pathogenesis, they may present with similar clinical and radiologic findings. Schiffner et al. found that in more than 20% of patients initially suspected of aseptic loosening, were ultimately diagnosed with a periprosthetic joint infection [28]. However, this study did not make such distinction due to its specific objectives.

An advantage of [^68^Ga]Ga-DOTA^Zol^ is the production of the radionuclide using ^68^Ge/^68^Ga-generator systems. Gallium-68 is generated from the decay of germanium-68 and can be extracted by elution under acidic conditions. Considering the half-lives (gallium-68: 68 min, germanium-68: 270.9 d), a secular equilibrium is reached and long generator operating times result [21]. Therefore, [^68^Ga]Ga-DOTA^Zol^ can be produced in a cost-effective manner in centers with access to a ^68^Ge/^68^Ga-generator system. In contrast, the production of ^18^flouride requires a cyclotron and is thus limited to a few highly specialized centres within Germany or needs a distribution system from the production site to the clinical centres. In times of increasing popularity of PET/CT diagnostics and the need for a continuous and cost-efficient radionuclide supply, generator-production could ensure a reliable radionuclide resource, even for smaller centres [21]. Nevertheless, the topics of gallium-68 radiolabelling and quality control still require further research and continuous improvement [29].

In summary, our findings support the use of [^68^Ga]Ga-DOTA^Zol^ in the context of periprosthetic loosening. The clinical scan protocol is practical, providing images with good spatial resolution. Regarding the methodological approach in our current study, sensitivity seems to be slightly lower compared to other methods and especially to [^18^F]fluoride PET/CT, whereas specificity is outstanding.

In addition to previously mentioned limitations – such as sample size and patient heterogeneity, diagnostic variability, scan timing, tracer-specific enrichment and differentiation of loosening types – it is important to highlight the retrospective study design with its limited statistical power. Hence, future research should focus on randomized, prospective studies with a larger patient collective. In this context, our research group is particularly focusing on the differentiation between septic and aseptic loosening and is investigating uptake kinetics through dynamic studies.

## Supplementary Information

Below is the link to the electronic supplementary material.

Supplementary file1 (PDF 675 KB)

## Data Availability

Data supporting the findings of this study are available within the article and its supplementary materials.
